# The Lepidoptera of Cuatrociénegas Protected Area 1. A new species in the genus *Callistege* Hübner, [1823] (Erebidae, Erebinae, Euclidiini) from the Chihuahuan Desert, Coahuila, Mexico

**DOI:** 10.3897/zookeys.1044.59773

**Published:** 2021-06-16

**Authors:** Nicholas T. Homziak, David C. Lightfoot, Eric H. Metzler, Kelly B. Miller

**Affiliations:** 1 Department of Biology and Museum of Southwestern Biology, University of New Mexico, MSC03 2020, Albuquerque, NM 87131–0001, USA University of New Mexico Albuquerque United States of America; 2 U.S.N.M. Natural History Museum; Museum of Southwestern Biology, P.O. Box 45, Alamogordo, NM 88311-0045, USA University of Florida Gainesville United States of America; 3 Entomology and Nematology Department, P. O. Box 110620, University of Florida, Gainesville, FL 32611-0620, USA U.S.N.M. Natural History Museum Alamogordo United States of America

**Keywords:** Biological diversity hotspot, *Callistege clara*, desert wetlands, gypsum dunes geologic formation, White Sands

## Abstract

A new species of *Callistege* Hübner, [1823] (Lepidoptera, Erebidae, Erebinae, Euclidiini) is described from Cuatrociénegas Protected Area and Biosphere Preserve in Coahuila, Mexico. Adult male and female moths are illustrated, including genitalia. *Callistege clara* Homziak & Metzler, **sp. nov.** is one of 27 new species of insects discovered during an inventory survey of arthropods of White Sands National Monument, USA, and Cuatrociénegas Protected Area (Mexico), funded by the U.S. National Park Service. The Cuatrociénegas Basin is known for high endemism of aquatic and wetland biota within the Chihuahuan Desert. *Callistege clara* Homziak & Metzler, **sp. nov.** was found in a wetland environment.

## Introduction

Poole (1989) listed three species of *Callistege* Hübner, [1823] (Lepidoptera, Erebidae, Erebinae, Euclidiini), from the New World. The three species, *C. intercalaris* (Grote, 1882), *C. diagonalis* (Dyar, 1889), and *C. triangula* (Barnes & McDunnough, 1918) were described from the southwestern United States; however, the distribution of these species in Mexico is not documented. The species described here was collected as part of an arthropod inventory of the Cuatrociénegas Protected Area, Coahuila, México (Cuatrociénegas), from 2010 through 2012 (Lightfoot et al. 2017). The project was sponsored by the U.S. National Park Service (NPS) Sister Parks Program, in cooperation with the Comisión Nacional de Áreas Naturales Protegidas, representing Cuatrociéngas. The overall study was an inventory of arthropods of Cuatrociénegas and White Sands National Monument, now White Sands National Park, New Mexico, U.S.A. (White Sands). The inventory leading to this discovery was conducted by personnel from the University of New Mexico, Museum of Southwestern Biology, Division of Arthropods (MSBA). The arthropod survey of the sister parks White Sands National Monument (Schneider-Hector 1993) and Cuatrociénegas Protected Area was initiated with the goal of discovering arthropod species endemic to these biodiversity hotspots (Schnieder 2011). Both parks, with soils high in gypsum content leading to distinctively white substrates in the extensive dune regions, are in the Chihuahuan Desert. The Cuatrociénegas Protected Area is located in a basin which possesses year-round fresh water in deep pools supplied by springs (Smith et al. 2011). These parks are home to numerous endemic moth species (Metzler 2014, 2016, 2017a, b; Metzler and Landry 2016; Metzler and Lightfoot 2014; Metzler and Porter 2018; Metzler and Scott-Tracey 2019, Metzler et al. 2009, 2010a, b, 2016; Metzler and Forbes 2011; Wright 2014) supposedly adapted to the high gypsum content of their environment or for the isolated patches of lush vegetation surrounding the pools where *C. clara* was collected.

Cuatrociénegas was declared a Natural Protected Area in 1994, and as a United Nations Educational, Scientific and Cultural Organization (UNESCO), Biosphere Preserve in 2004. Cuatrociénegas Biosphere Preserve is managed by the office of the Área de Protección de Flora y Fauna Cuatrociénegas under the direction of the Comisión Nacional de Áreas Naturales Protegidas, a unit of the Secretaría de Medio Ambiente y Recursos Naturales (Secretary for the Environment-and Natural Resources). We adopt the naming and spelling as used by the Área de Protección de Flora y Fauna Cuatrociénegas: Cuatrociénegas Protected Area.

White Sands and Cuatrociénegas both represent extensive landscapes of gypsum and saline soils including gypsum dunes, and both are in Chihuahuan Desert basins surrounded by uplifted limestone fault-block mountains. White Sands has the largest gypsum dune field in the world (1000 km^2^) and little surface water, while Cuatrociénegas has a smaller (20 km^2^) gypsum dune field, with extensive natural springs, ponds, lakes streams, marshes, and other wetlands. The MSBA conducted a series of field trips to Cuatrociénegas in 2010 and 2011 to sample arthropods, particularly targeting undescribed species. A wide variety of arthropods were sampled in the survey and reported elsewhere (Lightfoot et al. 2017). The series of a new species of *Callistege* that was collected from a wetland in the Cuatrociénegas Protected Area is reported here.

The primary landscape of Cuatrociénegas includes gypsum/salt flats, gypsum dunes, and many surface water features, including ephemeral lakes or playas, permanent springs, ponds, lakes, streams, and canals. Many of the surface water features were altered by humans such that canals drain most of the springs and ponds. Lower piedmont alluvial slopes are present, with rocky and gravelly soils. The gypsum dune areas of Cuatrociénegas cover approximately 20 square kilometers and were historically impacted by mining of the gypsum and grazing of vegetation by livestock. The principal vegetation types in the basin include halophytic and gypsum adapted associations, mesquite shrublands, rosetofilous/succulent (*Agave*, *Yucca*, *Acacia*, *Prosopsis*, *Larrea*, and cacti) shrublands, microphyllus desert shrublands, and a variety of anthropogenic vegetation types including irrigated and non-irrigated croplands. All landscape environments of Cuatrociénegas are impacted by humans, primarily from water diversions for agricultural use outside of the basin, and intense year-round domestic livestock (cattle, horses, burros) grazing throughout the basin, with concentrations adjacent to open water. Cuatrociénegas is particularly well known for the extensive aquatic environments and for supporting a large array of oases surrounded by desert and mountains. The spring-fed surface waters are unusually low in nutrients, especially nitrogen and phosphorous, and support unique and ancient microbial communities including stromatolites (Souza et al. 2012).

The type locality (Site C7, MSBA sampling location) is a popular site for visitors called Río Mezquites, a large freshwater stream and associated ponds and wetlands. The *Callistege* specimens were collected at the edge of a marsh with tall grass surrounding a deep pool adjacent to Río Mezquites.

## Materials and methods

Adult moths were collected over the course of one night using a 175W self-ballasting mercury vapor lamp reflected off of a white sheet as described in Covell (2005). The collecting site was selected to provide an opportunity to collect flying insects living in the isolated stream, pool, and wetland communities in Cuatrociénegas.

Genitalia were examined following procedures outlined in Clarke (1941), Hardwick (1950), Lafontaine (2004), and Pogue (2002). Abdomens were removed, wetted in ethanol, soaked in KOH, dissected, stained in Chlorazol Black in water, Orcein in 2-Propanol, dehydrated in 2-Propanol, and slide mounted in Euparal.

Wing pattern terminology follows Barnes and McDunnough (1918) and Lafontaine (2004), and genital structure terminology follows Lafontaine (2004), Klots (1970), and Goater et al. (2003). Forewing lengths, from the base to the apex excluding fringe, were measured to the nearest 0.1 mm using a stereo microscope fitted with a reticle.

Specimens of Lepidoptera cited in this study are deposited in the following collections:

**MSBA** Museum of Southwestern Biology, Division of Arthropods, University of New Mexico, Albuquerque, New Mexico (K.B. Miller, curator)

**USNM**United States National Museum of Natural History (Smithsonian Institution), Washington, DC (P. Z. Goldstein, curator)

## Taxonomic account

### Callistege clara

Taxon classificationAnimaliaLepidopteraErebidae

Homziak & Metzler sp. nov.

7B57F5CA-F74B-5855-9EE2-4B1F60A39147

http://zoobank.org/958EA223-4911-4518-B3CF-7B63571BE7D2

**Figure 1.**
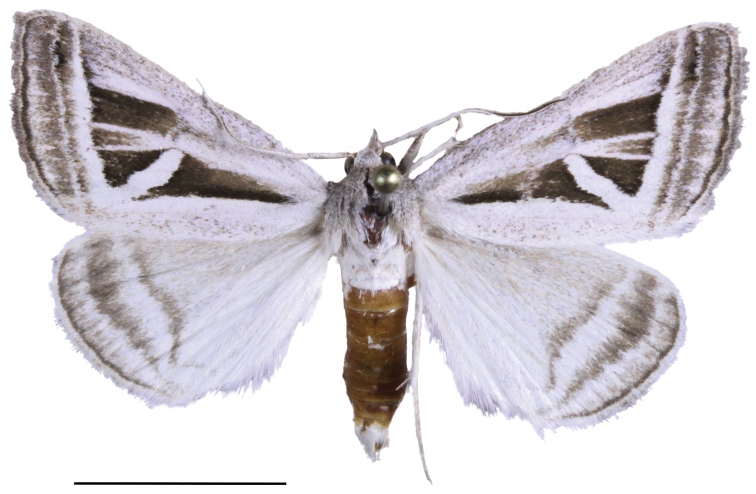
*Callistege clara* Homziak & Metzler, sp. nov., male (holotype). Mexico, Cuatrociénegas (MSBA 72901). Scale bar: 10 mm.

**Figure 2.**
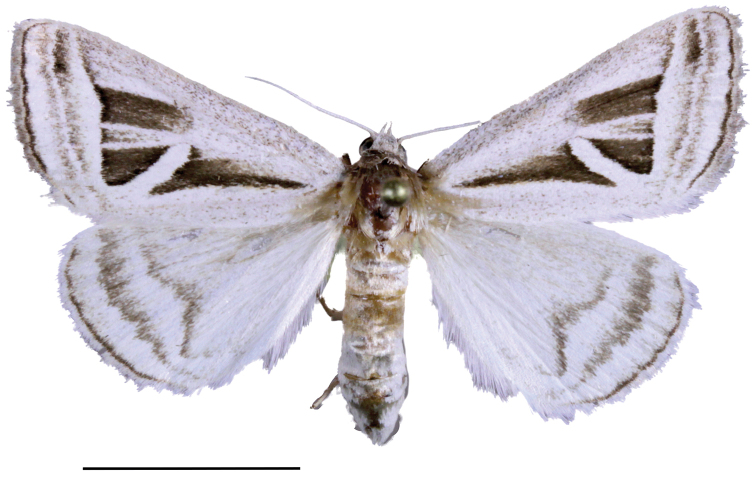
*Callistege clara* Homziak & Metzler, sp. nov., female (paratype). Mexico, Cuatrociénegas (MSBA 72903). Scale bar: 10 mm.

**Figure 3.**
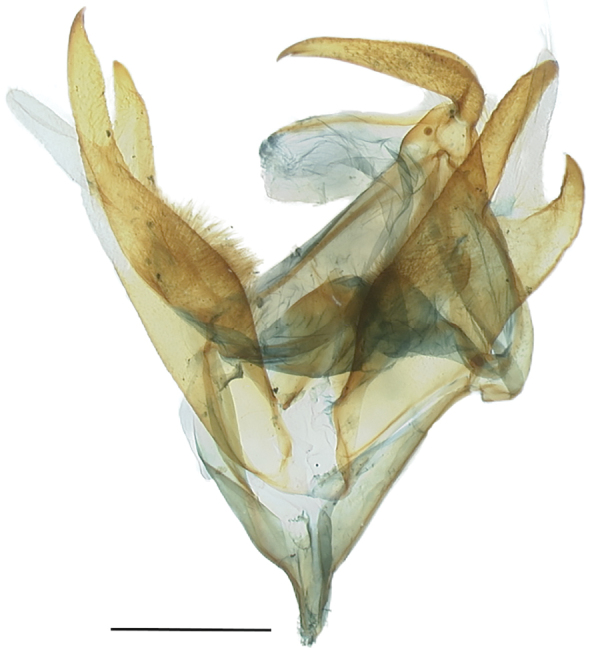
*Callistege clara* Homziak & Metzler, sp. nov. Mexico, Cuatrociénegas (paratype), male genitalia. Slide E.H.M. 585 (USNM). Scale bar: 1 mm.

**Figure 4.**
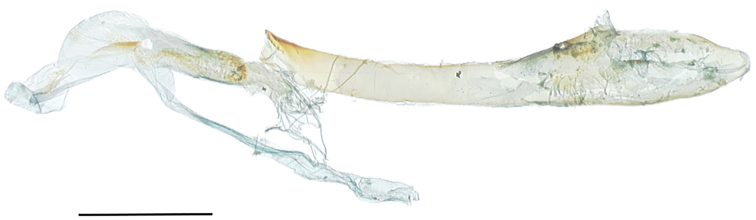
*Callistege clara* Homziak & Metzler, sp. nov. Mexico, Cuatrociénegas (paratype), aedeagus. Slide E.H.M. 586 (USNM). Scale bar: 1 mm.

**Figure 5.**
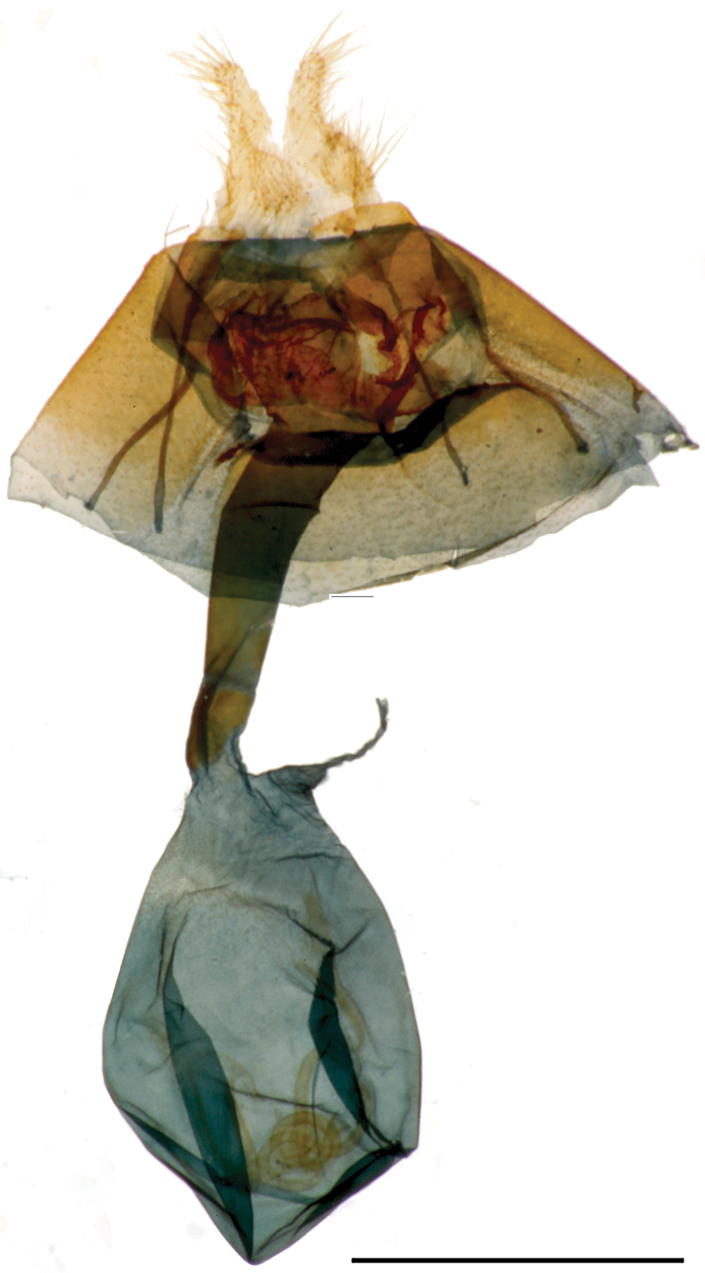
*Callistege clara* Homziak & Metzler, sp. nov. Mexico, Cuatrociénegas (paratype), female genitalia. Slide E.H.M. 587 (USNM). Scale bar: 2 mm.

Figures 1 , 2 , 3 , 4 , 5

#### Material.

***Holotype***: adult male, pinned: **Mexico: “Coahuila**: Cuatro Cienegas [sic] Nat[iona]l Preserve, 18-IX-2011, 26.91851°N 102.10211°W, D.C. Lightfoot et al. col[lecto]rs; Site C7, Río Mezquites, basin floor ponds streams, general collecting” MSBA 72901. **Paratypes**: 2 males, slide numbers E.H.M. 585, E.H.M. 586 (USNM) and 3 females, slide number E.H.M. 587 (USNM), MSBA 72902, slide number E.H.M. 588, MSBA 72903 (MSBA): MEXICO: “Coahuila: Cuatro Cienegas [sic] Nat[iona]l Preserve, 18-IX-2011, 26.91851°N 102.10211°W, D.C. Lightfoot et al. col[lecto]rs; Site C7, Río Mezquites, basin floor ponds streams, general collecting.”

#### Diagnosis.

**Figure 6.**
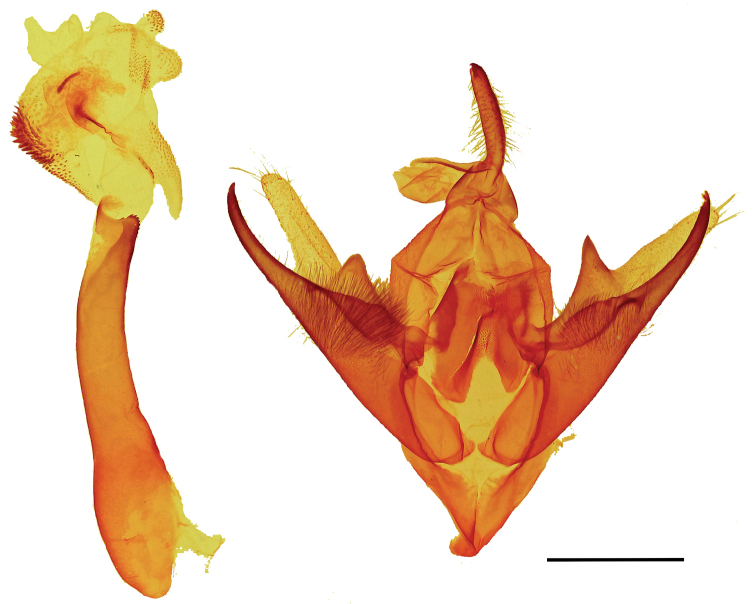
*Callistege triangula* male genitalia. JGF slide number 6533. John G. Franclemont Slide Collection, Cornell University. Scale bar: 1 mm.

**Figure 7.**
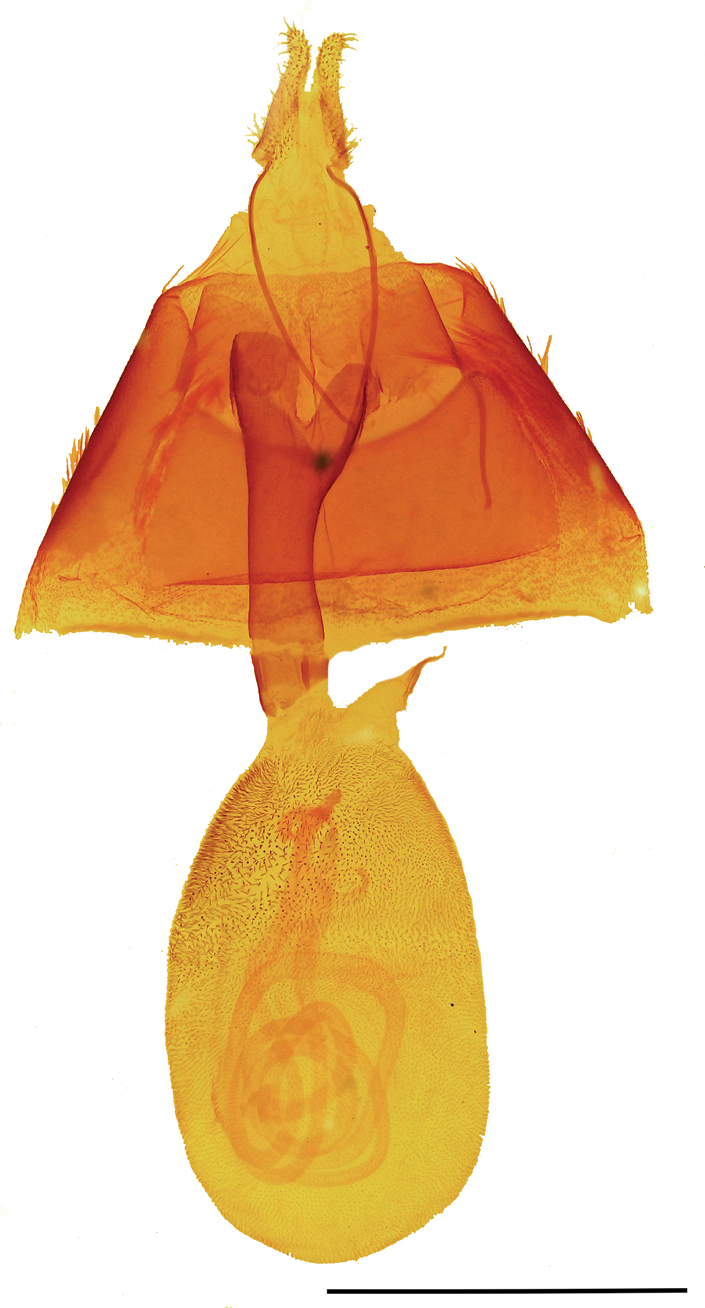
*Callistege triangula* female genitalia. JGF slide number 6534. John. G. Franclemont Slide Collection, Cornell University. Scale bar: 2 mm.

*Callistege clara* adults (Figs 1, 2) are easily recognized. The ground color of the forewing is white with two conspicuous triangular patches of dark brown, one conspicuous polygon patch of dark brown, and inconspicuous gray and dark brown markings. The hind wing is white with gray-brown postmedial, subterminal, and terminal lines. The ground color of the other three species in the genus is gray and brown; males and females of all four species respectively are similar. The male and female genitalia of *C. clara* (Figs 3–5) are markedly asymmetrical. In comparison, the respective genitalia of *C. triangula* (Figs 6, 7) are less asymmetrical. The dorsal margin of the valvae forms a hooked, claw-like lobe in *C. clara*; in *C. triangula*, these structures come to a peak. In *C. clara*, the sterigma is complex and sclerotized, versus simple and lightly sclerotized sterigma of *C. triangula. Callistege triangula* has spicules on the corpus bursae, whereas *C. clara* does not have spicules on the corpus bursae.

#### Description.

All three males and all three females in the type series are the basis for the following descriptions. ***Adult male*** (Fig. 1). *Head*: front smooth, scales appressed, dark brown tipped with cream; vertex scales rough proximally; labial palpi stout, porrect, scales appressed, similar to rest of head, but lighter color, basal segment and second segment wide, laterally flattened, third segment 0.4× length and width of second segment; haustellum well developed; antenna fasciculate, dorsoventrally flattened, oval cross section, dorsal surface scales cream, appressed, ventral surface naked, numerous sensory setae. *Thorax*: dorsum: anterior scales appressed, dark brown, cream tipped, posterior scales light brown, lateral scales long, hair-like, tegulae scales mixed spatulate and long, hair-like; venter white, scales mixed lamellar and piliform; fore leg: femurs with elongate, light brown and cream lamellar scales, tibia, epiphysis spatulate, tarsomeres with two rows of spines on ventral surface, terminal claws, mid leg: femurs with light brown spatulate scales, tibia and tarsomeres with two rows of spines, one pair of tibial spurs at distal end, tarsomeres spined on ventral surface; hind leg: femur scales spatulate, dorsal surface scales brown and cream, ventral surface white, two pairs of tibial spurs, tarsomeres ventrally spined, three rows; forewings: length 16.0–17.0 mm, mean 16.6 mm, n = 3; dorsal surface white and cream, scattered brown scales; costal margin base to postmedial line white, posterior margin base to tornus white, diagonal line from mid costa to tornus white, streak from base to diagonal mark white, broad, filling discal area, parallel white streaks from white diagonal line, along M2 and M3 to postmedial line, postmedial line two elements: basal element white, broad, second element gray, subterminal line three elements: basal element white, middle element gray, terminal element white with gray intrusions on the veins, terminal line black, three triangle marks, dark red brown, basal and dorsad of white posterior margin to curved white line, distal of curved white line and dorsad of white posterior margin, sub-costal and distal of curved white line, fringe brown, becoming cream towards tornus, underside a mixture of brown and white scales, appearing a dirty cream color, darker at costal and terminal areas, terminal lines same as above, discal spot crescent-shaped, brown, with streak extending basally from center, together forming a “Y” shape; Hind wings white, dusted with darker scales, postmedial line brown, not sharply defined, fringe cream colored, underside color as FW, apically fewer brown scales, becoming white towards the anal area, medial, subterminal, and terminal lines faintly visible, fringe concolorous. *Abdomen*: scales appressed, color light cream; underside similar. *Male genitalia* (Fig. 3): tegumen with lightly sclerotized lens-shaped region surrounded by lateral heavier sclerotization; juxta moderately sclerotized with a rough texture; vinculum narrow at base, widening distally, vaguely Y shaped, outer margins dark and sclerotized, becoming clear and more membranous mesially; valvae asymmetrical; saccular region smooth at base, with raised, setose patch in middle; cucullar region sparsely setose distally, distal end on each side divided in to two claw-like lobes, membranous projection originating between lobes on dorsal surface, larger of the lobes on left side with a pointed tip, right side with a more rounded tip; uncus setose, pointed downward at tip; aedeagus (Fig. 4): slightly curved, slightly expanded at base, sclerotized at distal end, vesica with patch of fine cornuti.

***Adult female*** (Fig. 2). Similar to male, forewings: length 16.0–17.5 mm, mean 16.6 mm, n = 3. Female genitalia (Fig. 5): anterior apophyses ca. 2/3 length of posterior apophyses; sterigma asymmetrical, complex, approximately trapezoidal forming two peaked corners anterior to ostium bursae, small bumps on surface; ostium bursae approximately circular; antrum more sclerotized on left side with numerous small bumps and spicules; ductus bursae sclerotized, narrowing anteriorly towards corpus bursae; corpus bursae membranous, narrowed at insertion of ductus bursae; appendix bursae small, projecting from right side of corpus bursae at base; posterior apophyses widened, flattened, wavy at tip; papillae analis membranous, setose, setae stout, variable length.

#### Distribution and biology.

This species is described from a wetland area adjacent to Río Mezquites, in a gypsum/limestone desert basin in the Cuatrociénegas Protected Area of the Coahuila de Zaragoza state in Mexico. The larva and its food plant(s) are unknown.

#### Etymology.

The specific epithet, *clara*, is chosen because of the much lighter ground color of this species relative to other members of this genus. It is treated as a singular adjective.

#### Remarks.

This species is placed in the genus *Callistege* on the basis of the following features: light colored angled bands over a darker ground color, a forewing pattern shared with other North American members of the genus, and the bifurcated valvae of the male genitalia.

While *C. clara* can clearly be distinguished from the other three North American species in the genus, our research revealed that the identity of *C. intercalaris* may be uncertain. Grote (1882) described *C. intercalaris* from an unknown number of specimens collected by Professor Snow in “New Mexico.” Todd (1982) stated the existence of two syntypes of *C. intercalaris*; one at the Natural History Museum, London (BMNH) and the other in the Snow Collection at the University of Kansas in Lawrence, KS (SEMC). Todd suggested the two syntypes represent two species. Poole (1989) stated the holotype of *C. intercalaris* was in the BMNH. Examination of specimens at SEMC, and photographs of the the type specimen at the BMNH demonstrated that *C. clara* is distinct in color and wing pattern from syntpes of *intercalaris*.

Careful attempts to evert the vesica of two male syntypes of *C. clara* were unsuccessful. Because the differences noted in the diagnosis separate *C. clara* from the other described species of *Callistege* of the Nearctic, prudence dictated that we leave the abdomen of the third male, the holotype, intact.

## Discussion

One objective of this research was to detect and describe new arthropod species, especially those endemic to White Sands or Cuatrociénegas. No comprehensive surveys of terrestrial and aquatic arthropods were conducted at Cuatrociénegas prior to the 2010–2011 MSBA inventory study, yet numerous endemic arthropods species, mostly crustaceans and scorpions, were reported from Cuatrociénegas prior to this study. Specifically, Dinger et al. (2005) conducted a survey of aquatic invertebrates from a number of springs, ponds, lakes, and streams at Cuatrociénegas, but did not report finding any new or endemic species. However, Cole (1984) had reported 12 species of aquatic crustaceans were known from Cuatrociénegas, six of which were endemic to Cuatrociénegas, including two recently described endemic genera (*Paramexiweckelia* Holsinger and *Sphaerolana* Cole & Minckley) consisting of six newly described species. These two genera were the only aquatic arthropod taxa known to be endemic to Cuatrociénegas prior to the MSBA inventory study.

In addition to endemic aquatic arthropods, a number of endemic terrestrial arthropods also are known from Cuatrociénegas. Cazier (1982) described two new species of endemic apiocerid flies from Cuatrociénegas: *Apiocera minkleyi* Cazier and *A. bigelowi* Cazier. Six species of scorpions were described from Cuatrociénegas, and five of those species are apparently endemic. Williams (1968) described five species of scorpions of the genus *Vaejovis* (*V. gilvus* Williams, *V. pallidus* Williams, *V. casieri* Williams, *V. coahuilae* Williams, and *V. minckleyi* Williams) from the Cuatrociénegas basin, and all but *V. coahuilae* appear to be endemic to the Cuatrociénegas basin. Soleglad (1974) described another endemic species of *Vaejovis* (*V. calidus* Soleglad) from Cuatrociénegas. Haradon (1985) described the scorpion *Paruroctonus coahuilanus* Haradon from Cuatrociénegas, and the species also appears to be endemic there. In total, eight species of scorpions were described from and are currently known to occur only in the Cuatrociénegas basin.

The MSBA arthropod inventory study discovered 22 new species of terrestrial arthropods at Cuatrociénegas, including; three grasshoppers (Orthoptera), three katydids (Orthoptera), three crickets (Orthoptera), one cockroach (Blattodea: Corydiidae; *Arenivaga gumperza*, Hopkins 2014), six other beetles (Coleoptera), two moths (Lepidoptera) and one spider (Araneae) (Lightfoot et al. 2017). Additionally, two of the new species at Cuatrociénegas also represent one new genus of katydid, and one new family of spider, which was recently described (Araneae: Myrmecicultoridae: Entelygynae: *Myrmecicultor chihuahuensis*; Ramirez et al. 2019). Two of the other six new species of beetles found in the UNM inventory survey include three darkling beetles (Tenebrionidae), two that were recently described (Smith et al. 2011, Wirth and Smith 2017), two species of scarab beetles described by Smith and Paulson (2017), and three new species of elaterid click beetles that were recently described (Johnson and Lightfoot 2018).

Two years of limited field surveys at Cuatrociénegas that focused only on certain arthropod groups for which there was taxonomic expertise available. This strongly indicates that many more undescribed arthropods remain to be discovered at Cuatrociénegas, especially among the Diptera, Hymenoptera, Coleoptera and Hemiptera, groups that were not fully identified in that study due to a lack of taxonomic expertise.

## Supplementary Material

XML Treatment for Callistege clara
